# Histone deacetylase inhibitor MGCD0103 causes cell cycle arrest, apoptosis, and autophagy in liver cancer cells

**DOI:** 10.7150/jca.34091

**Published:** 2020-01-29

**Authors:** Bo Liao, Quan Sun, Yufeng Yuan, Yuchun Yin, Jianguo Qiao, Ping Jiang

**Affiliations:** Department of Hepatopancreatobiliary Surgery, Zhongnan Hospital of Wuhan University, Wuhan 430071, China

**Keywords:** liver cancer, MGCD0103, cell growth, cell cycle arrest, apoptosis, autophagy

## Abstract

**Background:** Liver cancer is a common cause of cancer-related death all over the world. MGCD0103, a histone deacetylase inhibitor, exerts antitumor effect on various cancers. However, its role in liver cancer remains unclear.

**Methods:** The effect of MGCD0103 on HepG2 and Huh7 cells was verified by several experiments such as cell viability assay, colony formation assay, cell cycle analysis, apoptosis analysis, reactive oxygen species (ROS) assay, western blotting, immunohistochemistry, and xenograft assay.

**Results:** Cell viability and colony formation assays showed that MGCD0103 inhibited the proliferation of liver cancer cells *in vitro*. Flow cytometry and western blotting analysis demonstrated that MGCD0103 induced G2/M phase arrest and mitochondrial-related apoptosis. A pan-caspase inhibitor and ROS scavenger inhibited apoptosis induced by MGCD0103. What's more, MGCD0103 led to autophagy associated with cell death and an autophagy inhibitor inhibited apoptosis and autophagy induced by MGCD0103. Ultimately, MGCD0103 attenuated tumor growth but did not show significant systemic toxicity in animal model.

**Conclusions:** MGCD0103 suppressed the growth of liver cancer cells *in vitro* and *in vivo*. It could serve as a novel therapeutic approach for liver cancer.

## Introduction

Liver cancer is one of the leading causes of cancer-related deaths worldwide and China alone accounts for about half of the total number of cases and deaths 1. Liver cancer at early stages might be curable by liver transplantation or surgical resection 2. However, there are still no curative treatments for advanced liver cancer due to its resistance to chemotherapy. Sorafenib have shown survival benefits for patients with advanced liver cancer 3. However, it is not widely used in Asia due to the expensive cost and increased adverse events 4, 5, and therefore, novel therapeutic drugs need to be developed for advanced liver cancer.

Genome instability and mutation are one of the hallmarks of cancer 6. Inactivation of tumor suppressor genes can be acquired through epigenetic mechanisms such as histone modifications 7. Histone acetyl transferase (HAT) and histone deacetylase (HDAC) are the two enzymes controlling the level of acetylation of histones 8. HDAC removes acetyl groups from histone, causes gene silencing and exerts a pro-oncogenic effect. The balance between HAT and HDAC activity is disrupted in many cancers. HDAC inhibitors repress the activity of HDAC enzymes and therefore exert anticancer activity. HDAC inhibitors promote acetylation of histones and non-histone protein substrates. The HDAC enzymes are the targets of the HDAC inhibitors and grouped into four types. Class I HDAC enzymes include HDAC1, HDAC2, HDAC3, and HDAC8. Class II HDACs include HDAC4, HDAC5, HDAC6, HDAC7, HDAC9, and HDAC10. Class III HDACs, also named sirtuins, include seven human isoenzymes (SIRT 1-7). Class IV HDAC is HDAC11.

**Figure 1:**
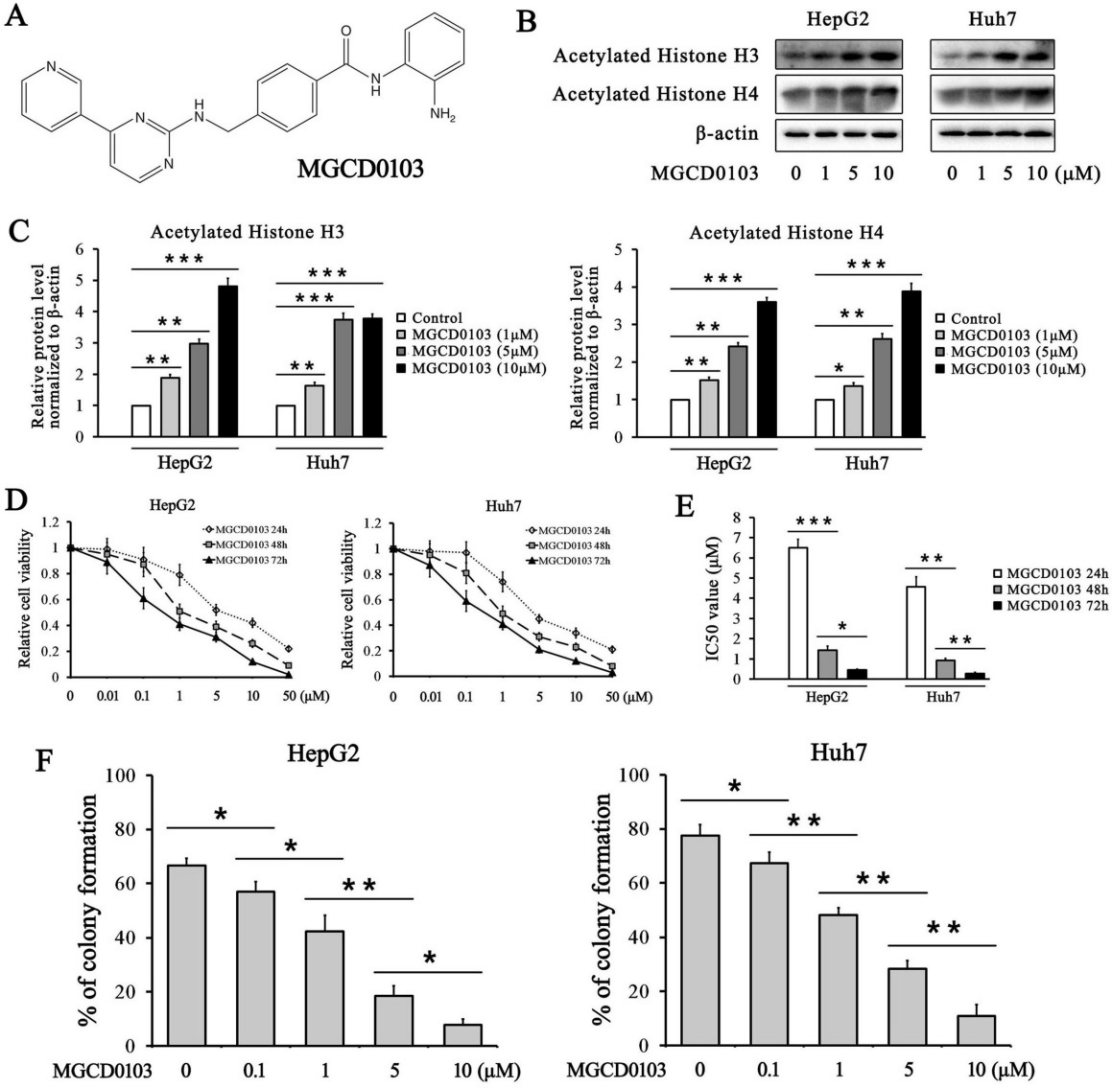
** MGCD0103 upregulates acetylated histone H3/4 and inhibits the growth of liver cancer cells in a dose-dependent manner. (A)** The chemical structure of MGCD0103. **(B, C)** HepG2 and Huh7 cells were treated with increasing concentrations of MGCD0103 for 48 h, and the expression of acetylated histone H3 and H4 was detected by western blotting. **(D)** HepG2 and Huh7 cells were treated with increasing concentrations (0.01, 0.1, 1, 5, 10, and 50μM) of MGCD0103 for 24 h, 48 h, or 72 h. Cell viability was assessed using the CCK-8 assay. **(E)** The IC50 values of MGCD0103 in HepG2 and Huh7 cell lines were calculated. **(F)** Colony formation assay. HepG2 and Huh7 cells were treated with different concentrations (0.01, 1, 5, and 10μM) of MGCD0103. The percentage of colonies formed was calculated. *P < 0.05; **P < 0.01; ***P < 0.001

Several HDAC inhibitors such as vorinostat, romidepsin, belinostat, and panobinostat have been approved by the FDA for the treatment of cancer 9. After that, more and more HDAC inhibitors are in different phases of clinical trials and MGCD0103 (mocetinostat) is one of them. MGCD0103, shown in Fig. 1A, was developed in Canada by MethylGene 10. It is a synthesized HDAC inhibitor, highly specific for classes I and IV HDACs. This compound selectively inhibits HDAC1, HDAC2, HDAC3 and HDAC11. It was studied in Phase I/ II trials for solid tumors and hematologic malignancies 11-13. Preclinical studies have demonstrated that it can induce apoptosis and cell cycle blockade in various kinds of cancer 10. Other studies have shown that it can induce or suppress autophagy in different cancers 14, 15. However, its role in the treatment of liver cancer remains still unclear.

In this study, we explored the effect of MGCD0103 on growth, apoptosis, cell cycle, and autophagy in liver cancer cells *in vitro* and *in vivo*. The related molecular mechanisms were also investigated.

## Materials and Methods

### Cell lines and cell cultures

HepG2 (a hepatoma cell line) and Huh7 (a hepatocellular carcinoma cell line) cell lines were purchased from China Center for Type Culture Collection (CCTCC, Wuhan, China). Cell lines were maintained in high-glucose Dulbecco's modified Eagle's medium (DMEM) (Gibco, NY, USA) containing 10% fetal bovine serum (FBS) (Gibco, NY, USA). Cells were cultured at cell culture incubator with 37 °C and 5% CO2.

### Drugs and reagents

MGCD0103, Z-VAD-FMK, and 3-Methyladenine (3-MA) were purchased from Selleck Chemicals (TX, USA). Crystal violet, 5-FU, N-Acetyl Cysteine (NAC), and DMSO were purchased from Sigma-Aldrich (MO, USA). Cell Counting Kit-8 (CCK-8) was purchased from Dojindo (Kumamoto, Japan).

### Cell viability assay

HepG2 (1×10^3^ cells/well) and Huh7 (1.5×10^3^ cells/well) cells were plated in 96-well plates and treated with increasing concentrations (0.01, 0.1, 1, 5, 10, and 50μM) of MGCD0103 for different lengths of time (24 h, 48 h, and 72 h) to generate cell viability curves. CCK-8 reagent was added for 2 h and the optical density (OD) values were measured at 450nm. The half maximal inhibitory concentration (IC50) was calculated by SPSS software.

### Colony formation assay

Colony formation assay was carried out as described previously 16. HepG2 (500 cells/well) and Huh7 (500 cells/well) cells were plated in 6-well plates and treated with different concentrations (0.1, 1, 5, and 10 μM) of MGCD0103 for 48 h. Media were refreshed every other day. The plates were stained with crystal violet and the images were acquired on day 14. The numbers of colonies were counted and analyzed by Alpha Innotech Imaging system (Alphatron Asia Pte Ltd, Singapore).

### Western blotting

Western blotting was performed as described previously 17. The primary antibodies against acetylated histone H3, acetylated histone H4, and β-actin were purchased from Santa Cruz Biotechnology (CA, USA). The primary antibodies against p21, p27, cdc25C, cyclin B1, Bim, Bax, Bcl-2, Bcl-xL, RAGE, and LC3 were purchased from Epitomics (CA, USA). The primary antibodies against p-cdc25C, cdc2, p-cdc2, Cyto-C, cleaved caspase-9, cleaved caspase-3, cleaved caspase-7, cleaved-PARP, PARP, PI3KC3, Beclin 1, and p62 were purchased from Cell Signaling Technology (MA, USA). All secondary antibodies were purchased from Jackson Immuno Research Laboratories (PA, USA).

### Cell cycle and apoptosis analysis

The flow cytometry analysis was carried out as described previously 18. For cell cycle analysis, HepG2 and Huh7 cells were treated with MGCD0103 for 48 h. A total of 1×10^6^ cells were analyzed by FACSAria Cell Cytometer (BD Biosciences, CA, USA). For apoptosis analysis, 1×10^5^ cells were analyzed. All data were analyzed by CellQuest software (BD Biosciences, CA, USA).

### ROS assay

The ROS assay was performed as described previously 19. HepG2 and Huh7 cells were treated with MGCD0103 in the presence or absence of NAC (5 mM) for 48 h. The fluorescent probe DCFH-DA was used to evaluate intracellular ROS levels. Cells were analyzed by FACSAria Cell Cytometer.

### Animal model

Animal experiments were performed as previously described 16, 20. All procedures in animal experiments were approved by the Committee on the Ethics of Animal Experiments of Zhongnan Hospital, Wuhan University. HepG2 cells were subcutaneously injected into the mice. MGCD0103 treatment started on day 9 after injection when the tumors were palpable. MGCD0103 was dissolved in DMSO and dosed per os (p.o.) daily. All the animals were sacrificed by cervical dislocation on day 24.

Subcutaneous tumors were dissected, removed and conserved for proliferation and apoptosis analysis. AST, ALT, Cr, and BUN levels in mice blood were measured by Reflotron Plus system (Roche, IN, USA). Organ tissues including heart, liver, spleen, lung, and kidney were removed and sectioned.

### Immunohistochemistry analysis

Immunohistochemistry was performed as previously described 16, 20. Ki-67 primary antibody was purchased from Dako (Golstrup, Denmark). Tumor tissues were incubated with primary antibodies against Ki-67 and cleaved caspase-3, then incubated with a secondary antibody. Organ tissues were stained with H&E for the histological analysis.

### Statistical analyses

Statistical analyses were performed by SPSS 13.0 ( IL, USA). All experiments were carried out at least three independent times. The results were expressed as the mean ± SD. Comparisons between the different groups were analyzed by one-way ANOVA, and *P* < 0.05 was considered to be statistically significant.

## Results

### MGCD0103 increases the acetylation of histone H3 and H4 in liver cancer cell lines

The effect of MGCD0103 on the acetylation of histone H3 and histone H4 was evaluated in HepG2 and Huh7 cells. The western blotting results showed that treatment with increasing concentrations of MGCD0103 for 48 h increased the acetylation level of histone H3 and histone H4 in HepG2 and Huh7 cell lines in a dose-dependent manner (Fig. 1B and C).

### MGCD0103 suppresses the growth of liver cancer cells

To investigate the inhibitory effect of MGCD0103 on liver cancer cells, HepG2 and Huh7 cell lines were treated with MGCD0103. The CCK-8 assay demonstrated that MGCD0103 exhibited dose-dependent and time-dependent cytotoxic effects on HepG2 and Huh7 cells (Fig. 1D and E). The IC50 values of MGCD0103 in HepG2 cells for different lengths of time (24 h, 48 h, and 72 h) were 6.497 ± 0.431 μmol/L (μM), 1.427 ± 0.206 μM, and 0.453 ± 0.055 μM, respectively, and those in Huh7 cells were 4.567 ± 0.496, 0.920 ± 0.096, and 0.277 ± 0.061μM, respectively (Fig. 1E). The results indicated that MGCD0103 exerted anti-proliferative activity against liver cancer cells. Colony formation assay showed that MGCD0103 reduced the colony numbers of HepG2 and Huh7 cells in a dose-dependent manner (Fig. 1F). The colony formation rates of HepG2 cells treated with increasing concentrations (0, 0.1, 1, 5, and 10 μM) of MGCD0103 were 66.54 ± 2.71%, 56.91 ± 3.68%, 42.37 ± 5.93%, 18.41 ± 3.76%, and 7.72 ± 2.15%, respectively, and those in Huh7 cells were 77.50 ± 4.03, 67.22 ± 4.02, 48.25 ± 2.65, 28.38 ± 3.01, and 10.86 ± 4.20%, respectively (Fig. 1F).

### MGCD0103 induces cell cycle arrest in liver cancer cells

**Figure 2:**
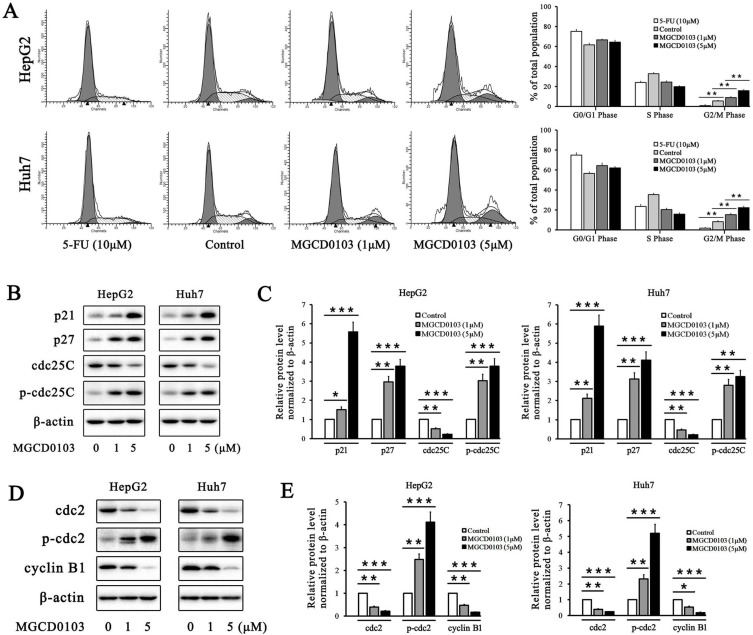
** MGCD0103 causes G2/M phase arrest in liver cancer cells. (A)** HepG2 and Huh7 cells were treated with 5-FU (10 μM) and MGCD0103 (1 μM and 5μM) for 48h. Cell cycle distribution was then assessed using flow cytometry. **(B-E)** Western blotting analysis of p21, p27, cdc25C, p-cdc25C, cdc2, p-cdc2, and cyclin B1 after MGCD0103 treatment. *P < 0.05; **P < 0.01; ***P < 0.001

5-FU, as the positive control, caused cell cycle arrest in HepG2 and Huh7 cells at G0/G1 phase (Fig. 2A). The proportion of cells at G2/M phase was decreased after treatment with 5-FU (Fig. 2A). Compared with the control group, MGCD0103 caused G2/M cell cycle arrest in HepG2 and Huh7 cells (Fig. 2A). The proportions at G2/M phase of HepG2 cells treated with increasing concentrations (0, 1, and 5 μM) of MGCD0103 were 5.55 ± 0.58%, 8.90 ± 0.90%, and 15.72 ± 1.14%, respectively, and those of Huh7 cells were 8.16 ± 1.18, 15.26 ± 1.45, and 22.20 ± 1.72%, respectively (Fig. 2A). Some related proteins were tested by western blotting. MGCD0103 upregulated the protein levels of p21, p27, p-cdc25C, and p-cdc2, while downregulated those of cdc25C, cdc2, and cyclin B1 in a dose-dependent manner (Fig. 2b-e).

### MGCD0103 triggers apoptosis in liver cancer cells

**Figure 3:**
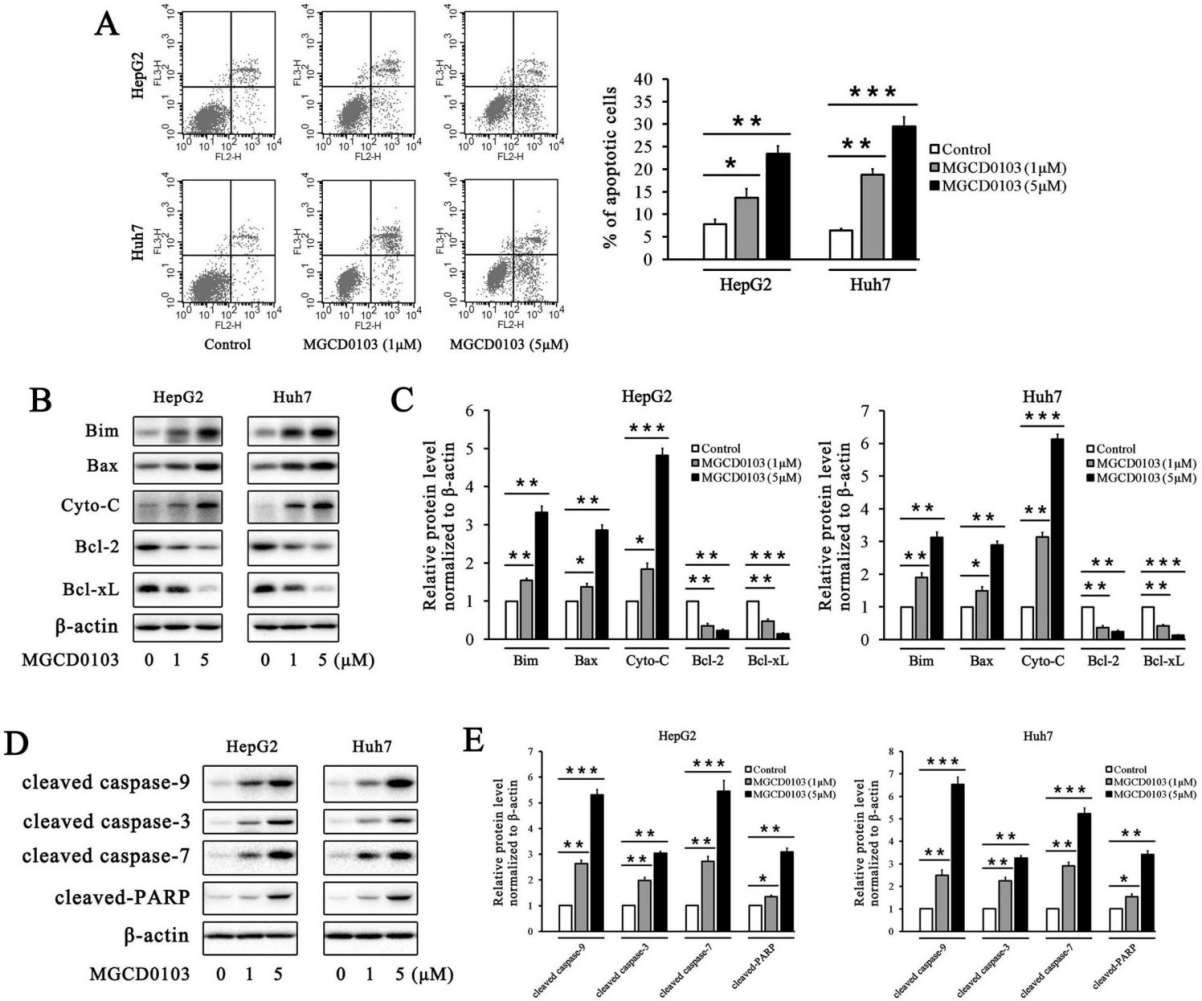
** MGCD0103 causes apoptosis in liver cancer cells. (A)** HepG2 and uh7 cells were treated with MGCD0103 (1 μM and 5μM) for 48h. Apoptosis was evaluated by flow cytometry. Apoptotic rate was then calculated. **(B-E)** Western blotting analysis of Bim, Bax, Cyto-C in cytosol, Bcl-2, Bcl-xL, cleaved caspase-9, cleaved caspase-3, cleaved caspase-7, and cleaved-PARP after MGCD0103 treatment. *P < 0.05; **P < 0.01; ***P < 0.001

The flow cytometry analysis showed that the apoptotic rates of HepG2 and Huh7 cells were elevated after treatment with MGCD0103 in a dose-dependent manner (Fig. 3a). The apoptotic rates of HepG2 cells treated with increasing concentrations (0, 1, and 5 μM) of MGCD0103 were 7.84 ± 1.03%, 13.63 ± 2.03%, and 23.47 ± 1.69%, respectively, and those of Huh7 cells were 6.45 ± 0.41, 18.78 ± 1.27, and 29.48±2.13%, respectively (Fig. 3A). Several apoptosis-related proteins were detected by western blotting. MGCD0103 downregulated the expressions of Bcl-2 as well as Bcl-xL, and upregulated those of Bim, Bax, Cyto-C, cleaved caspase-9, cleaved caspase-3, cleaved caspase-7, and cleaved-PARP in a dose-dependent manner (Fig. 3B-E).The above alterations indicated the activation of the mitochondria apoptosis pathway.

**Figure 4:**
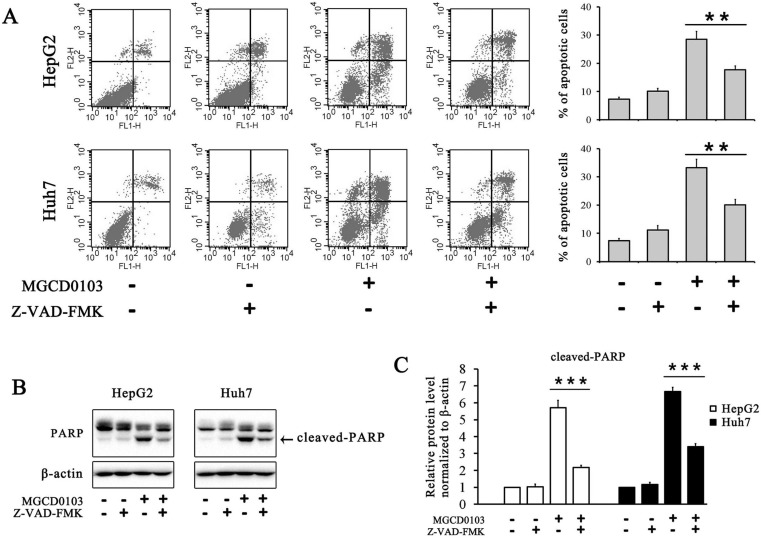
** Caspase inhibitor Z-VAD-FMK partially inhibited apoptosis induced by MGCD0103 in liver cancer cells. (A)** HepG2 and HUH7 cells were treated with 20 μM Z-VAD-FMK for 1 h and then 5μM MGCD0103 for the next 48 h. Apoptosis was evaluated by flow cytometry. **(B, C)** Western blotting analysis of cleaved-PARP after MGCD0103 and/or Z-VAD-FMK treatment. **P < 0.01; ***P < 0.001

To further evaluate the effect of MGCD0103 on the intrinsic apoptotic pathway, HepG2 and Huh7 cells were pretreated with the caspase inhibitor Z-VAD-FMK (20 μM) before treatment with MGCD0103. The pretreatment of Z-VAD-FMK decreased the apoptotic rate caused by MGCD0103 from 28.47 ± 2.85 to 17.74 ± 1.32% in HepG2 cells and from 33.29 ± 2.93 to 20.06 ± 2.02% in Huh7 cells (Fig. 4A). Western blotting showed that Z-VAD-FMK attenuated the cleavage of PARP induced by MGCD0103 (Fig. 4B and C).

### ROS participates in the apoptosis induced by MGCD0103 in liver cancer cells

**Figure 5:**
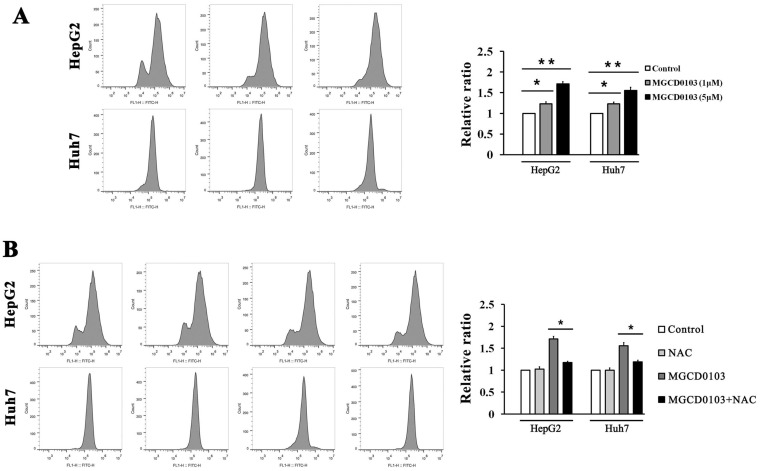
** ROS inhibitor NAC partially decreased MGCD0103- induced ROS levels in liver cancer cells.** First, HepG2 and Huh7 cells were treated with increasing concentrations (0, 1, and 5μM) of MGCD0103 for 48 h. Then cells were pretreated with 5mM NAC for 1 h and then treated with 5μM MGCD0103 for the next 48 h. **(A, B)** ROS levels were evaluated by flow cytometry.

**Figure 6:**
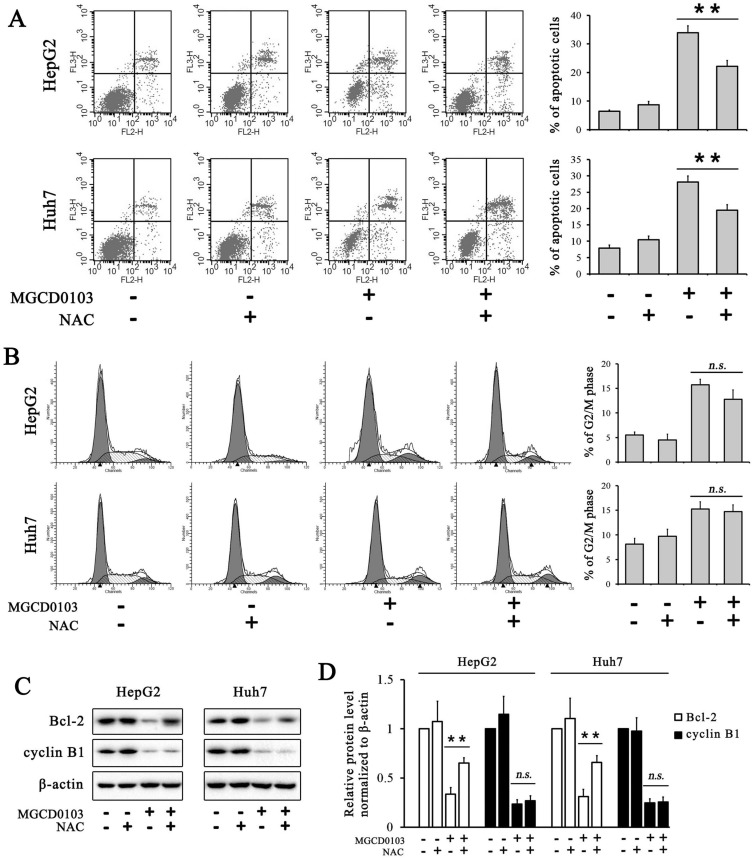
** ROS inhibitor NAC partially inhibited apoptosis induced by MGCD0103 in liver cancer cells.** Cells were pretreated with 5mM NAC for 1 h and then treated with 5μM MGCD0103 for the next 48 h. **(A)** Apoptosis was evaluated by flow cytometry. **(B)** Cell cycle distribution was evaluated by flow cytometry. **(C, D)** Western blotting analysis of Bcl-2 and cyclin B1 after MGCD0103 and/or NAC treatment. *P < 0.05; **P < 0.01

ROS produced by the mitochondria participates in the regulation of apoptosis, and inhibition of apoptosis by antiapoptotic Bcl-2 is related to the protection against ROS 21. The ROS assay demonstrated that ROS levels in HepG2 and Huh7 cells were dose-dependently increased after treatment with MGCD0103 (Fig. 5A). Cells were then pre-treated with NAC (5 mM) before treatment with MGCD0103 for a further 48 h. Pretreatment with NAC led to significantly decreased MGCD0103-induced ROS levels (Fig. 5B). HepG2 and Huh7 cells were pretreated with NAC (5mM) before treatment with MGCD0103 to verify if ROS may play a role in apoptosis or G2/M phase arrest induced by MGCD0103. The apoptotic effect was reversed by the addition of NAC which decreased the apoptotic rate from 28.09 ± 1.84% caused by MGCD0103 to 19.45 ± 1.74% in HepG2 cells and from 33.94 ± 2.43 to 22.19 ± 1.98% in Huh7 cells (Fig. 6A). However, NAC did not influence G2/M phase arrest caused by MGCD0103 (Fig. 6B). Western blotting showed that NAC restored the expression of Bcl-2 reduced by MGCD0103, but failed to impact on the decreased expression of cyclin B1 induced by MGCD0103 (Fig. 6C and D). The above results revealed that ROS was involved in the mitochondrial apoptosis but not in cell cycle arrest induced by MGCD0103.

### MGCD0103 results in autophagy correlated with cell death in liver cancer cells

**Figure 7:**
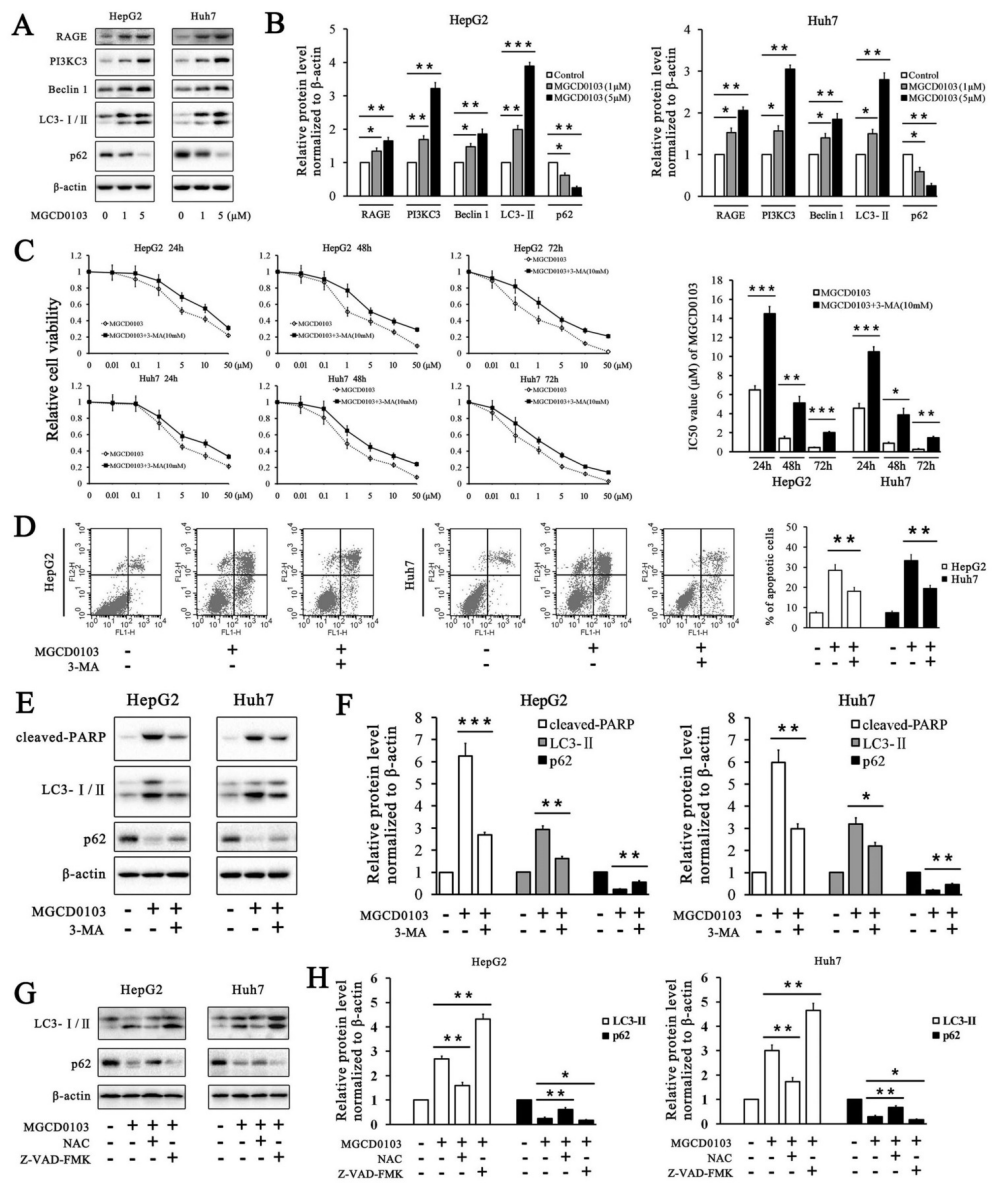
** MGCD0103 causes autophagy and autophagic cell death in liver cancer cells. (A, B)** HepG2 and HUH7 cells were treated with MGCD0103 (1 μM and 5μM) for 48h. Several autophagy-related proteins including RAGE, PI3KC3, Beclin 1, LC3-I/II, and p62 were then analyzed using western blotting. **(C)** HepG2 and Huh7 cells were treated with increasing concentrations (0.01, 0.1, 1, 5, 10, and 50μM) of MGCD0103 alone or in combination with 5 mM 3-MA for 24 h, 48 h, or 72 h. Cell viability was assessed using the CCK-8 assay. The IC50 values of MGCD0103 were calculated and compared. **(D)** HepG2 and Huh7 cells were treated with 5 μM MGCD0103 alone or in combination with 5 mM 3-MA for 48 h. Apoptosis was evaluated by flow cytometry. **(E, F)** HepG2 and Huh7 cells were treated with 5 μM MGCD0103 alone or in combination with 5 mM 3-MA for 48 h. The protein level of cleaved-PARP, LC3-I/II, and p62 was tested by western blotting. **(G, H)** HepG2 and Huh7 cells were treated with MGCD0103 (5 μM) in the presence or absence of NAC (5 mM) and Z-VAD-FMK (20 μM) for 48 h. The protein level of LC3-I/II and p62 was tested by western blotting. *P < 0.05; **P < 0.01; ***P < 0.001

It has been reported that two HDAC inhibitors OSU-HDAC42 and SAHA induced autophagy in liver cancer cells and SAHA-induced autophagy led to cell death 22. We found that MGCD0103, one of HDAC inhibitors, could also induce autophagy in HepG2 and Huh7 cells. Several autophagy-related proteins were tested by western blotting. MGCD0103 increased the expressions of RAGE, PI3KC3, Beclin 1, and LC3-II, and decreased that of p62 in a dose-dependent manner (Fig. 7A and B). The relative cell viability of HepG2 and Huh7 cells was significantly higher after exposure to 3-MA (a PI3KC3 inhibitor blocking autophagosome formation) and MGCD0103 than after treatment with MGCD0103 alone (Fig. 7C).

Addition of 3-MA (5 mM) increased the IC50 values of MGCD0103 in HepG2 cells for different lengths of time from 6.497 ± 0.431 to 14.497 ± 0.732 (24 h), from 1.427 ± 0.206 to 5.120 ± 0.700 (48 h), and from 0.453 ± 0.055 to 2.037 ± 0.111 μM (72 h), respectively (Fig. 7C). Consistent with the effect of 3-MA on HepG2 cells, 3-MA also increased the IC50 values of MGCD0103 in Huh7 cells from 4.567 ± 0.496 to 10.490 ± 0.520 (24 h), from 0.920 ± 0.096 to 3.887 ± 0.669 (48 h), and from 0.277 ± 0.061 to 1.470 ± 0.142 μM (72 h), respectively (Fig. 7C). Apoptosis analysis showed that addition of 3-MA decreased the apoptotic rate from 28.47 ± 2.85% caused by MGCD0103 to 18.08 ± 1.88% in HepG2 cells and from 33.29 ± 2.93 to 19.53 ± 1.55% in Huh7 cells (Fig. 7D). Western blotting revealed that 3-MA reduced the cleavage of PARP, accumulation of LC3-II induced by MGCD0103, and degration of p62 induced by autophagy (Fig. 7E and F). We then investigated the effect of Z-VAD-FMK and NAC on autophagy. Western blotting showed that whereas NAC attenuated accumulation of MGCD0103-induced, autophagy-related proteins, Z-VAD-FMK exposure increased MGCD0103-induced, autophagy-related proteins (Fig. 7G and H).

### MGCD0103 inhibits tumor growth in animal model

**Figure 8:**
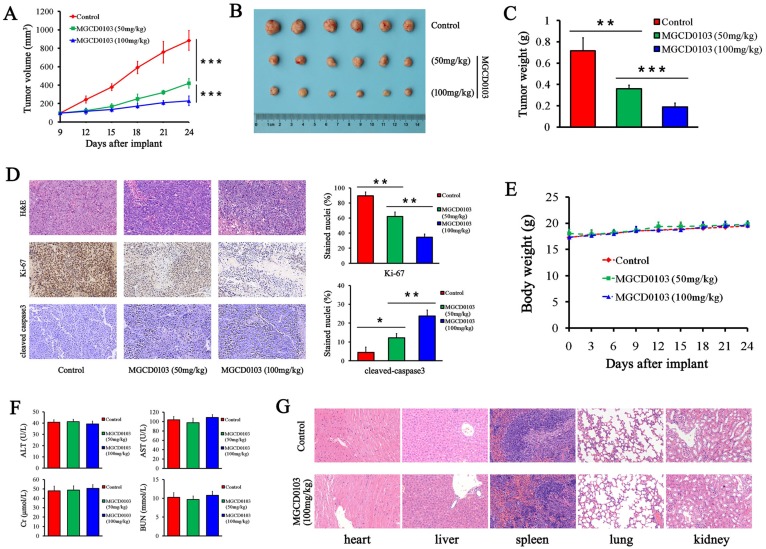
** MGCD0103 inhibited tumorigenicity *in vivo*. (A)** The tumor growth curve of HepG2 cells. MGCD0103 treatment was initiated 9 days after tumor implantation. Tumor volume was calculated every 3 days. Mice were randomized into three groups (n = 6) and MGCD0103 was dosed per os (p.o.) daily at 50 or 100 mg/kg. **(B)** Representative images of the subcutaneous tumors in the three groups. **(C)** Tumor weights in the different groups were measured and compared. **(D)** Representative images of H&E, Ki-67, and cleaved caspase-3 staining of tumor sections. The percentage of Ki-67 and cleaved caspase-3 positive cells was calculated and compared in the different groups. **(E)** The body weight curve of mice in the three groups. Body weight was measured every 3 days. **(F)** The levels of AST, ALT, Cr, and BUN of mice blood were detected by biochemistry analyzer. **(G)** Representative images of H&E staining of heart, liver, spleen, lung, and kidney. The histologic structures were observed and compared microscopically. *P < 0.05; **P < 0.01; ***P < 0.001

To further investigate the effect of MGCD0103 on liver cancer cell growth *in vivo*, HepG2 cells were subcutaneously injected into nude mice. MGCD0103 treatment was begun on the 9th day, and it was p.o.-administered daily at 50 or 100 mg/kg for 15 days. The results showed that MGCD0103 significantly suppressed the tumor volume (Fig. 8A and B). The tumor weight of MGCD0103-treated mice was much lighter than that of the control group (Fig. 8C). Ki-67 and cleaved caspase-3 staining of tumor sections was performed to evaluate tumor proliferation and apoptosis *in vivo*. MGCD0103 significantly decreased the percentage of Ki-67 positive cells, and significantly increased the percentage of cleaved caspase-3 positive cells (Fig. 8D).

Afterwards we evaluated the toxicity after MGCD0103 treatment. We found that MGCD0103 exerted no obvious effect on body weight of mice and biochemical functions of liver and kidney (Fig. 8E and F).

Organs including heart, liver, spleen, lung, and kidney were stained with H&E. Histological analysis showed no significant histological changes after MGCD0103 treatment (Fig. 8G).

## Discussion

Several HDAC inhibitors, including those approved by FDA, have shown anticancer activity in liver cancer; a few relevant clinical trials are ongoing 23, 24. Although MGCD0103 is a HDAC inhibitor, its effect on liver cancer remains unknown. Zhou and his colleagues carried out pharmacokinetic studies of orally administered MGCD0103 in mice, rats, and dogs 25. In their studies, they found that this compound had good oral bioavailability and was quickly absorbed across all species tested 25. What's more, MGCD0103 showed significant anticancer activity in several tumor xenograft models in a dose-dependent manner and a more potent antiproliferative effect than MS-275 and SAHA in a number of human cancer cell lines 10. In a phase 1 study in patients with hematologic malignancies, pharmacokinetic analyses demonstrated absorption of MGCD0103 within 1 hour after oral administration and a long elimination half-life in plasma of 9 plus or minus 2 hours 26. Another phase 1 study of MGCD0103 in patients with advanced solid tumors found that its elimination terminal half-life ranged from 6.7 to 12.2 hours and no accumulation after repeated dosing was observed 11. MGCD0103 undergoes hepatic metabolism and is eliminated via biliary and fecal routes 27.

**Figure 9:**
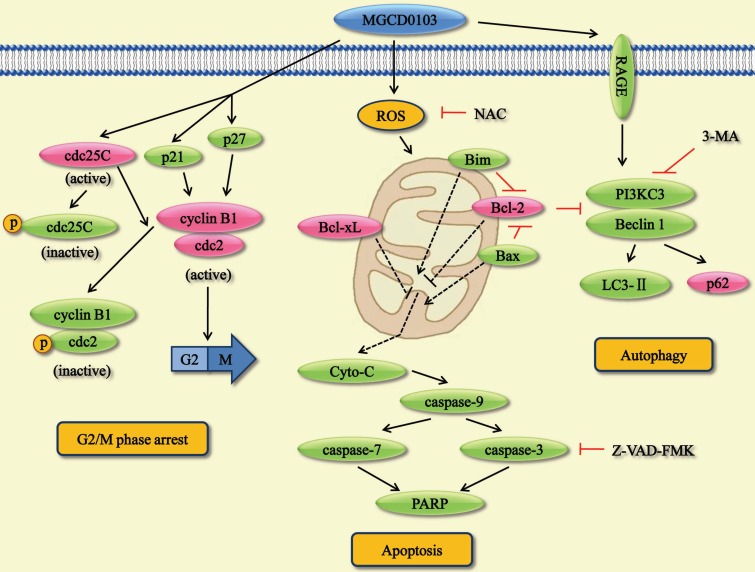
Overview of the pathways for MGCD0103-induced cell cycle arrest, apoptosis, and autophagy in liver cancer cells.

In the present study, we found that MGCD0103, an isotype-specific HDAC inhibitor, had anticancer activity in liver cancer cell lines. This compound augmented the acetylated level of histone H3 and histone H4 by using western blotting. Moreover, we demonstrated that MGCD0103 significantly inhibited tumor growth *in vivo* and *in vitro*. This agent caused G2/M phase cell cycle arrest, apoptosis, and autophagy by affecting a series of related proteins (Fig. 9). MGCD0103 did not lead to organ-related and systemic toxicities in mice.

The hallmark of G2/M phase arrest is the deactivation of cdc25C and cdc2/cyclin B1 complex 28. Cdc25C can dephosphorylate cdc2 and thus activate cdc2/cyclin B1 complex which promotes the G2/M transition. Phosphorylation of cdc25C and cdc2 reduces their activity and causes G2/M phase arrest. Furthermore, p21 and p27 can inhibit cdc2 activity to affect cell cycle 29, 30. In this study, cell cycle ananlysis showed that MGCD0103 resulted in G2/M arrest in a dose-dependent manner in HepG2 and Huh7 cells. Western blotting found that p21, p27, p-cdc25C, and p-cdc2 were upregulated and cdc25C, cdc2, and cyclin B1 were downregulated after treatment with MGCD0103. Changes in cell cycle-related proteins may induce G2/M phase arrest in MGCD0103-treated cells.

Apoptosis is a process of programmed cell death. Some factors like caspases induce apoptosis, while some members of the Bcl-2 family suppress apoptosis. Mitochondria apoptosis pathway is the major pathway involving in apoptosis. Mitochondria can produce ROS and release Cyto-C which activates its downstream caspases.

It has been reported that MGCD0103 exerts its anticancer activity by causing apoptosis in a variety of cancer cells 10, 31. In our study, we found that MGCD0103 triggered apoptosis in a dose-dependent manner in HepG2 and Huh7 cells. Western blotting showed that proapoptotic proteins like Bim and Bax were upregulated and antiapoptotic proteins like Bcl-2 and Bcl-xL were downregulated after treatment with MGCD0103. What's more, expression of Cyto-C in cytosol was elevated in MGCD0103-treated cells. Following the treatment with MGCD0103, a significant increase of cleavage of caspase-9, -3, -7, and PARP was also observed. The caspase inhibitor Z-VAD-FMK and the ROS inhibitor NAC partially attenuated MGCD0103-induced apoptosis. The above results suggested that mitochondria participated in the apoptotic process induced by MGCD0103.

Apart from apoptosis, autophagy is a major form of programmed cell death. Previous studies have found that, during the process of autophagy, the expression of the receptor for advanced glycation end products (RAGE), Beclin 1, PI3KC3, and LC3-II is increased 22, 32. Meanwhile, p62 can be degraded by autophagy 33. In the present study, we demonstrated that MGCD0103 elicited autophagy in HepG2 and Huh7 cells. Western blotting revealed the overexpression of RAGE, PI3KC3, Beclin 1, and LC3-II and the decreased expression of p62. 3-MA, the PI3KC3 inhibitor, partially reversed autophagy and autophagic cell death induced by MGCD0103.

Further study revealed that NAC reversed MGCD0103-induced autophagy, which indicated a link between ROS and autophagy. The complex interplay between autophagy and apoptosis depends on various cellular settings. Flow cytometric analysis and western blotting demonstrated that autophagy induced by MGCD0103 contributed to apoptosis in liver cancer cells; and that Z-VAD-FMK further increased MGCD0103-induced autophagy even more, indicating that, when apoptosis was inhibited, autophagic cell death was prominent in liver cancer cells. Consequently, in our study, both apoptosis and autophagy induced by MGCD0103 cooperatively led to liver cancer cell death, with autophagy serving as a back-up mechanism when apoptosis was defective. The mechanisms that underlie the crosstalk between apoptosis and autophagy warrant further investigation.

Although most HDAC inhibitors approved by FDA are nonselective, MGCD0103, an isotype-selective HDAC inhibitor, targets HDAC1, HDAC2, HDAC3 and HDAC11. Our results indicate that anticancer efficacy by a HDAC inhibitor did not require that it inhibit all HDAC isotypes. Inhibition of certain HDAC isoforms by MGCD0103 was enough for antitumor activity in this study, and suggests that clinical use of less-comprehensive HDAC inhibitors for liver cancer and other cancers might offer efficacy with less toxicity.

In addition to efficacy, safety is an increasing concern for HDAC inhibitors. MGCD0103 is a rationally designed and orally bioavailable HDAC inhibitor 25. Expressions of fewer genes are induced by MGCD0103, a benzamide HDAC inhibitor, than are induced by nonselective hydroxamate HDAC inhibitors, yet the efficacy of MGCD0103 in preclinical tumor models is maintained or even elevated 10. Possibly because of its slow-tight kinetic properties, the off-binding rate of MGCD0103 is very low, so that its inhibitory effect is prolonged after its removal. Therefore, patients can receive lower doses and less frequent dosing of MGCD0103 while its pharmacodynamic effect is still maintained 34.

In brief, MGCD0103 may have advantages over broad-spectrum HDAC inhibitors due to longer half-life, good oral bioavailability and greater therapeutic index.

## Conclusions

In summary, the present study investigated the molecular mechanisms underlying the anticancer effect of MGCD0103 on liver cancer cells. MGCD0103 could cause G2/M phase arrest via cdc25C/ cdc2/cyclinB pathway, trigger apoptosis via mitochondrial pathway, and induce autophagy via RAGE/PI3KC3/Beclin 1 pathway. Furthermore, MGCD0103 could significantly inhibit the growth of liver cancer *in vitro* and *in vivo*. Consequently, MGCD0103 may be a novel and efficacious agent against liver cancer.

## Abbreviations

HDAC: histone deacetylase
HAT: histone acetyl transferase
3-MA: 3-Methyladenine
NAC: N-Acetyl Cysteine
CCK-8: Cell Counting Kit-8
IC50: half maximal inhibitory concentration
RAGE: receptor for advanced glycation end products
